# Life Course Themes in Japanese Americans’ Dementia Care Profiles

**DOI:** 10.1093/geroni/igaa057.1763

**Published:** 2020-12-16

**Authors:** Juyoung Park, Kyong Hee Chee, Angelica Yeh

**Affiliations:** 1 University of Southern California, Los Angeles, California, United States; 2 Texas State University, San Marcos, Texas, United States; 3 Alzheimer’s Los Angeles, San Gabriel, California, United States

## Abstract

The “Faces of Caregiving” video project highlights the social and cultural aspects of dementia care as shared by seven Japanese Americans. We performed a content analysis of the narratives in the videos, and prominent themes included stigma, gender, resilience, and values placed on family and community. We also applied the life course perspective in examining each care trajectory and found the principles of historical time, linked lives, timing, and human agency to be not only relevant but also useful for a comprehensive understanding of the shared and unique care experiences of Japanese Americans. These results have significant practical implications in that they inform a conceptual template for developing similar video projects for other Asian ethnic groups in the United States. The video project can be replicated within an appropriate cultural context, and such a culturally sensitive approach can help better meet diverse ethnic minorities’ unmet needs for information and education. Part of a symposium sponsored by the Aging Among Asians Interest Group.

